# Unresectable stage III non-small cell lung cancer: could durvalumab be safe and effective in real-life clinical scenarios? Results of a single-center experience

**DOI:** 10.3389/fonc.2023.1208204

**Published:** 2023-07-04

**Authors:** Paolo Borghetti, Giulia Volpi, Giorgio Facheris, Gianluca Cossali, Eneida Mataj, Salvatore La Mattina, Navdeep Singh, Jessica Imbrescia, Marco Lorenzo Bonù, Davide Tomasini, Paola Vitali, Diana Greco, Michela Bezzi, Flavia Melotti, Mauro Benvenuti, Andrea Borghesi, Salvatore Grisanti, Michela Buglione di Monale e Bastia

**Affiliations:** ^1^Radiation Oncology Department, Azienda Socio Sanitaria Territoriale (ASST) Spedali Civili and University of Brescia, Brescia, Italy; ^2^Division of Pneumology, University Hospital Azienda Socio Sanitaria Territoriale (ASST) Spedali Civili, Brescia, Italy; ^3^Institute of Pathology, Azienda Socio Sanitaria Territoriale (ASST) Spedali Civili, Brescia, Italy; ^4^Thoracic Surgery, Department of Cardio-Thoracic Surgery, Azienda Socio Sanitaria Territoriale (ASST) Spedali Civili, Brescia, Italy; ^5^Department of Medical and Surgical Specialties, Radiological Sciences, and Public Health, Azienda Socio Sanitaria Territoriale (ASST) Spedali Civili and University of Brescia, Brescia, Italy; ^6^Medical Oncology Department, Azienda Socio Sanitaria Territoriale (ASST) Spedali Civili and University of Brescia, Brescia, Italy

**Keywords:** non-small cell lung cancer (NSCLC), stage III, durvalumab, chemo-radiotherapy (CRT), real-world data (RWD)

## Abstract

**Introduction:**

The standard of care for patients with unresectable stage III non-small cell lung cancer (NSCLC) is chemoradiotherapy (CRT) followed by consolidation durvalumab as shown in the PACIFIC trial. The purpose of this study is to evaluate clinical outcomes and toxicities regarding the use of durvalumab in a real clinical scenario.

**Methods:**

A single-center retrospective study was conducted on patients with a diagnosis of unresectable stage III NSCLC who underwent radical CRT followed or not by durvalumab. Tumor response after CRT, pattern of relapse, overall survival (OS) and progression-free survival (PFS), and toxicity profile were investigated.

**Results:**

Eighty-five patients met the inclusion criteria. The median age was 67 years (range 45–82 years). Fifty-two patients (61.2%) started sequential therapy with durvalumab. The main reason for excluding patients from the durvalumab treatment was the expression of PD-L1 < 1%. Only two patients presented a grade 4 or 5 pneumonitis. A median follow-up (FU) of 20 months has been reached. Forty-five patients (52.9%) had disease progression, and 21 (24.7%) had a distant progression. The addition of maintenance immunotherapy confirmed a clinical benefit in terms of OS and PFS. Two-year OS and PFS were respectively 69.4% and 54.4% in the durvalumab group and 47.9% and 24.2% in the no-durvalumab group (p = 0.015, p = 0.007).

**Conclusion:**

In this real-world study, patients treated with CRT plus durvalumab showed clinical outcomes and toxicities similar to the PACIFIC results. Maintenance immunotherapy after CRT has been shown to be safe and has increased the survival of patients in clinical practice.

## Introduction

Non-small cell lung cancer (NSCLC) accounts for approximately 85% of all types of lung cancer (1). Approximately one-third of patients have locally advanced (LA) disease at diagnosis and are not eligible for surgical resection (2, 3). Concurrent chemoradiotherapy (cCRT) has been the standard of care (SoC) for patients with unresectable stage III NSCLC over the years (3), but the introduction of durvalumab (Imfinzi^©^, AstraZeneca Inc.) as consolidation immunotherapy after definitive cCRT have drastically improved overall survival (OS) and progression-free survival (PFS), as reported by the results of the PACIFIC trial (4). The PACIFIC regimen is now adopted in clinical practice, and it is considered the SoC for patients with unresectable stage III NSCLC suitable for chemoradiotherapy with radical intent (4–6).

Based on data from the PACIFIC study, regardless of levels of PD-L1 expression, on 16 February 2018, the Food and Drug Administration approved durvalumab as consolidation therapy following effective cCRT for patients with unresectable stage III NSCLC (7). The European Medical Agency (EMA) and the Italian Agency for Drugs (Agenzia Italiana del Farmaco (AIFA)) approved durvalumab after cCRT and sequential chemoradiotherapy (sCRT) in the same group of patients but exclusively in the case of PD-L1 expression of at least 1% (8).

The safety profile and results of pivotal randomized clinical trials (RCTs) often diverge from those achieved in real-world practice because they are designed for highly selected patient populations due to strict eligibility criteria and always do not represent the range of patients seen in real-world practice (9).

This is a single-center retrospective series of patients with unresectable stage III NSCLC treated with cCRT or sCRT followed or not by durvalumab while the PACIFIC regimen arose as SoC in Italy (October 2018). The objectives of this real-life analysis are twofold: the first one is to explore and describe the reasons for accessing or rejecting durvalumab as maintenance in daily practice. The second one is to analyze the clinical features, tumor response to cCRT, the pattern of relapse, toxicity profiles, and the survival outcomes of patients treated with CRT in comparison with the PACIFIC study.

## Material and methods

This is a single-center, retrospective, and observational study including all patients with unresectable stage III NSCLC treated with cCRT or sCRT followed or not by durvalumab at Radiation Oncology Department of Spedali Civili and the University of Brescia between October 2018 and July 2022.

The inclusion criteria were histological diagnosis of NSCLC, stage III disease according to TNM American Joint Committee on Cancer (AJCC) 8th edition (10) and unresectable disease as defined after multidisciplinary discussion in the lung unit with thoracic surgeons, radiologists, medical oncologists, and pneumologists.

Eligible patients received curative CRT. The prescribed dose was 60 Gy in 30 fractions (2 Gy/fr) delivered with intensity-modulated radiation therapy (IMRT), volumetric modulated arc therapy (VMAT), or helical IMRT (H-IMRT). Patients underwent free-breathing four-dimensional computed tomography (CT) simulation for treatment planning on which the gross tumor volume (GTV) was contoured as reported by ESTRO ACROP guidelines (11). All patients had a diagnostic positron emission tomography scan (PET-CT) later co-registered with the simulation CT to guide target volume delineation. An internal target volume (ITV) was created by the deformation of the clinical target volume (CTV) contour from one breathing phase to the others using the treatment planning system (TPS) Velocity^©^. All patients received daily image-guided radiotherapy (IGRT) with cone-beam CT (CBCT) or megavoltage CT (MVCT).

All patients were treated with platinum-based doublet chemotherapy with cCRT (at least two cycles during radiotherapy and no more than one cycle before radiotherapy) or sCRT (radiotherapy started after at least three cycles of chemotherapy).

Maintenance immunotherapy (durvalumab) after cCRT or sCRT was prescribed for patients with PD-L1 expression ≥1%, free from disease progression after completion of CRT, without clinical history of primary/secondary immunodeficiency, active infection, and pulmonary toxicity after CRT higher or equal to grade 3 (G3; according to Common Terminology Criteria for Adverse Events (CTCAE) version 5.0) (12).

During follow-up, total body CT scans were commonly performed: every 3 months in the first 2 years and every 6 months in the following years, or more frequently when clinically indicated.

Tumor response was assessed according to Response Evaluation Criteria in Solid Tumors (RECIST version 1.1). Locoregional progression included all sites of relapse within the involved pulmonary lobe(s) and the hilar and mediastinal nodal stations. Distant metastasis included the other sites of progression, as well as pulmonary lesions absent at the onset. OS was defined as the time between the end of radiotherapy and death or last assessment of vital status, while PFS was defined as the time from the end of radiotherapy to disease progression (any site) or death or last follow-up. Follow-up was defined as the time from the end of radiotherapy to the last assessment of clinical status.

All reported adverse events (AEs) were recorded according to CTCAE version 5.0 (11). All lung toxicities have been reported. In particular, pneumonia was recorded if the pulmonary infection was confirmed by blood, sputum, or bronchoalveolar lavage culture. The other non-infectious lung toxicities, such as acute interstitial pneumonitis, interstitial lung disease, pneumonitis, and pulmonary fibrosis, were all included in the group of pneumonitis/radiation pneumonitis. The latter grouping was necessary due to the unfeasibility to distinguish the etiology of this pneumonitis in patients treated with either CRT or durvalumab.

Statistical analysis of the collected data provided a description of the numerical frequency and the percentage of the variables. The chi-square test and t-test were applied for correlations between categorical and continuous variables, respectively. Survival curves were calculated using the Kaplan–Meier method. Survival estimates were calculated at 1 and 2 years. Log-rank test was used for comparison between groups. All statistical analyses were conducted using Software IBM-SPSS^®^ ver. 26.0.1 (IBM SPSS Inc., Chicago, IL, USA). A p-value <0.05 was considered statistically significant.

The Ethics Committee reviewed and approved the study protocol (Protocol No. 4762, approved on 16 June 2021).

## Results

Eighty-five patients were retrospectively included in this analysis.

### Patient, pathological, and treatment features

The median age was 67 years (range 45–82 years), and 60 patients were male (70.6%). All patients had Eastern Cooperative Oncology Group—Performance Status (ECOG PS) of 0 or 1, and Charlson Comorbidity Index ranged between 3 and 9. Only six patients had never smoked; the median pack-year resulted in 45. Forty-five patients (52.9%) reported chronic obstructive pulmonary disease (COPD) as respiratory comorbidity (grade 3 for eight patients).

Adenocarcinoma and squamous cell carcinoma were the histological types in 55.3% and 37.6% of cases, respectively. A PD-L1 expression was observed in 67 cases (78.8%), and mutation status was known in 45 patients. Within this group, 10 patients presented an oncogenic driver mutation; EGFR was mutated in 2.3% of patients.

All of the patients received PET-CT, only three patients had brain MRI, and 55 patients (64.7%) underwent endobronchial ultrasound (EBUS) as mediastinal staging.

Thirty-six (41.4%), 42 (49.4%), and seven patients (8.2%) were staged as IIIA, IIIB, and IIIC, respectively. The median volume of planning target volume (PTV) was 439 cc, ranging between 169 and 1171 cc. Most of the patients were treated with the VMAT technique.

All patients received 60 Gy, and the median overall treatment time was 42 days.

Forty-four patients (51.8%) received chemotherapy with a 3-weekly schedule, and the most-used drug combination was carboplatin and paclitaxel doublet. Seventy-four patients (87.1%) had a cCRT, and 11 patients received sCRT. No statistical differences in terms of clinical, pathological, and treatments were detectable between the groups of patients treated with or without durvalumab, except for PD-L1 expression (**Table 1**).

**Table 1:** Patient, histological and treatment features.

		All		CRT		CRT+durvalumab		p-Value
		Median	Min–max	Median	Min–max	Median	Min–max	
**Age (years)**		68	45−82	68	45−81	69	50−82	-
**Charlson Comorbidity Index**		5	3−9	5	3−9	6	3−9	–
**Pack years**		45	0−150	45	0−120	50	0−150	-
**PTV (cc)**		439	168.9−1,170.7	481	168.9−1,170.7	432	174−1,150	–
		N	%	N	%	N	%	
**Sex**	Male	60	70.6	21	63.6	39	75.0	0.262
	Female	25	29.4	12	36.4	13	25.0	
**Age (years)**	<65 years	32	37.6	12	36.4	20	38.5	0.789
	65–75 years	38	44.7	14	42.4	24	46.2	
	>75 years	15	17.6	7	21.2	8	15.4	
**ECOG**	0	47	55.3	20	60.6	27	51.9	0.432
	1	38	44.7	13	39.4	25	48.1	
**Educational status**	Primary school	28	32.9	10	30.3	18	34.6	0.946
	Secondary school	32	37.6	13	39.4	19	36.5	
	High school	21	24.7	8	24.2	13	25.0	
	Graduation	4	4.7	2	6.1	2	3.8	
**Smoking status**	Current	43	50.6	21	63.6	22	42.3	0.154
	Former	36	42.4	10	30.3	26	50.0	
	Never	6	7.1	2	6.1	4	7.7	
**COPD**	No	40	47.1	18	54.5	22	42.3	0.390
	Grade 1	19	22.4	7	21.2	12	23.1	
	Grade 2	18	21.2	7	21.2	11	21.2	
	Grade 3	8	9.4	1	3.0	7	13.5	
**Histology**	Adenocarcinoma	47	55.3	18	54.5	29	55.8	0.328
	Squamous cell carcinoma	32	37.6	11	33.3	21	40.4	
	Other	6	7.1	4	12.1	2	3.8	
**Mutations detected**	Mutational status known	48	56.5	21	24.7	27	31.8	0.238
	EGFR	2	2.3	0	0.0	2	2.4	
	KRAS	7	8.2	3	3.5	4	4.7	
	ALK	0	0	0	0.0	0	0.0	
	ROS1	2	2.3	0	0.0	2	2.4	
	MET	1	1.2	0	0.0	1	1.2	
**PD-L1 expression**	Not evaluated or 0	18	21.2	17	51.5	1	1.9	<0.00001
	1%–50%	35	41.2	5	15.2	30	57.7	
	>50%	32	37.6	11	33.3	21	40.4	
**Stage (sec. WHO VIII ed.)**	IIIA	36	41.4	12	36.4	24	46.2	0.672
	IIIB	42	49.4	18	54.5	24	46.2	
	IIIC	7	8.2	3	9.1	4	7.7	
**Treatment**	Concurrent	74	87.1	27	81.8	47	90.4	0.664
	Sequential	11	12.9	6	18.2	5	9.6	
**Chemo schedule**	Weekly	41	48.2	14	42.4	27	51.9	0.393
	3-weekly	44	51.8	19	57.6	25	48.1	
**Chemo type**	Carboplatin–paclitaxel	70	82.3	26	78.8	44	84.6	0.686
	Cisplatin–etoposide	3	3.5	1	3.0	2	3.8	
	Other	12	14.2	6	18.2	6	11.5	
**RT technique**	VMAT	80	94.1	33	100.0	47	90.4	0.079
	TOMO	5	5.9	0	0.0	5	9.6	

CRT, chemoradiotherapy; PTV, planning target volume; ECOG, Eastern Cooperative Oncology Group; COPD, chronic obstructive pulmonary disease; RT, radiation therapy; VMAT, volumetric modulated arc therapy; TOMO, tomotherapy.

### CRT response and sequential immunotherapy

A total body CT scan was performed for all patients to evaluate tumor response after CRT. A complete response (CR) was achieved in 2 cases, while partial response (PR) and stable disease (SD) were reported in 41 and 31 cases, respectively. Eight patients showed progression of disease (PD) at the CT scan. Three patients were not evaluated for the decline of clinical conditions (**Table S1** in the **Supplementary Material**).

Fifty-two patients (61.2%) started maintenance immunotherapy with durvalumab. Two patients received durvalumab within the expanded access program (EAP). The main reasons for exclusion from durvalumab treatment were the negative expression of PD-L1 in 13 patients (15.3%) and disease progression in eight patients (9.4%). Only two patients did not receive durvalumab because of G3 pulmonary toxicity after CRT (**Table 2**).

**Table 2:** Reasons for exclusion from durvalumab.

	N	(%)
**PD-L1 < 1%**	13	15.3
**Progression disease**	8	9.4
**Death**	3	3.5
**CRT pulmonary toxicity**	2	2.4
**History of autoimmune pathology**	1	1.2
**Other**	6	7.1
**All**	33	38.8

CRT, chemoradiotherapy.

The median time elapsed between the end of CRT and the start of durvalumab amounted to 47 days (ranging between 2 and 105 days). Seven patients underwent a new biopsy after CRT, and only in two cases did this lead to a positive expression of PD-L1.

Ten patients (19.2%) and 22 patients (42.3%) had respectively a temporary and definitive interruption in the group treated with durvalumab. Of the latter, the interruption was related to PD in 15 patients and severe toxicity in six patients, and one patient died of COVID-19. The median time of treatment with durvalumab was 46 weeks (ranging between 5 and 74 weeks).

### Pattern of recurrence and survivals

After a median follow-up of 20 months, 45 patients (52.9%) showed PD. Within this group, the pattern of recurrence was distant metastasis in 21 cases (46.6%), locoregional failure in seven cases (15.6%), and both distant and locoregional in 17 cases (37.8%). Twelve patients (14.1%) had bone metastasis, 11 patients (12.9%) presented brain metastasis, and seven patients (8.2%) had a local recurrence in the ipsilateral lung.

Locoregional recurrences, distant metastasis, and total progression events resulted higher in the group that did not receive durvalumab, but these differences were not statistically significant (p = 0.797, p = 0.506, and p = 0.509, respectively). The cumulative death rate at the end of follow-up was 36.5% for patients who received durvalumab (median follow-up 21 months) and 51.5% for patients not treated with immunotherapy (median follow-up 11 months), p = 0.031.

**Figure 1:**
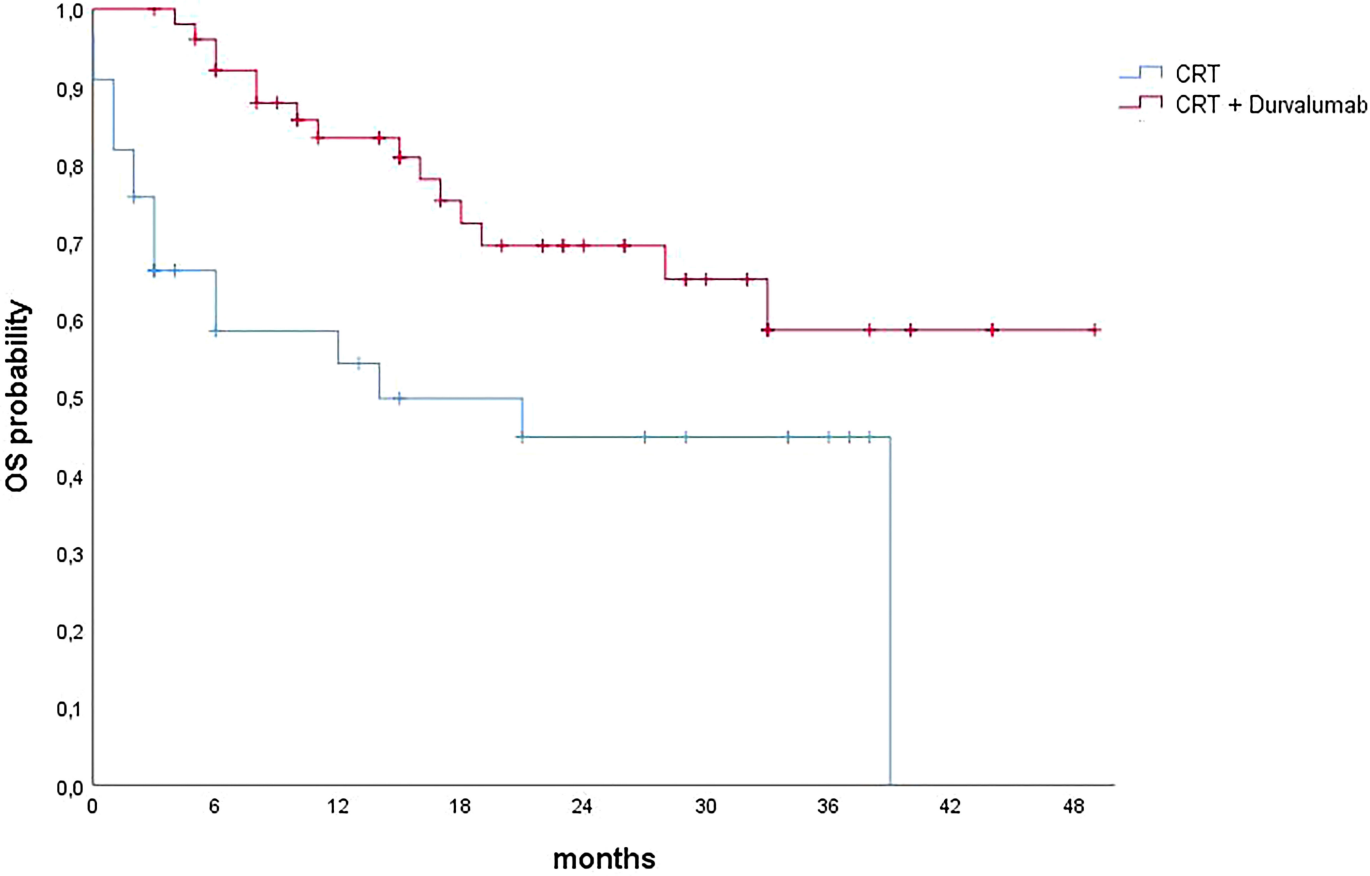
Overall survival curves calculated using the Kaplan–Meier method. CRT, chemoradiotherapy; OS, overall survival.

**Figure 2:**
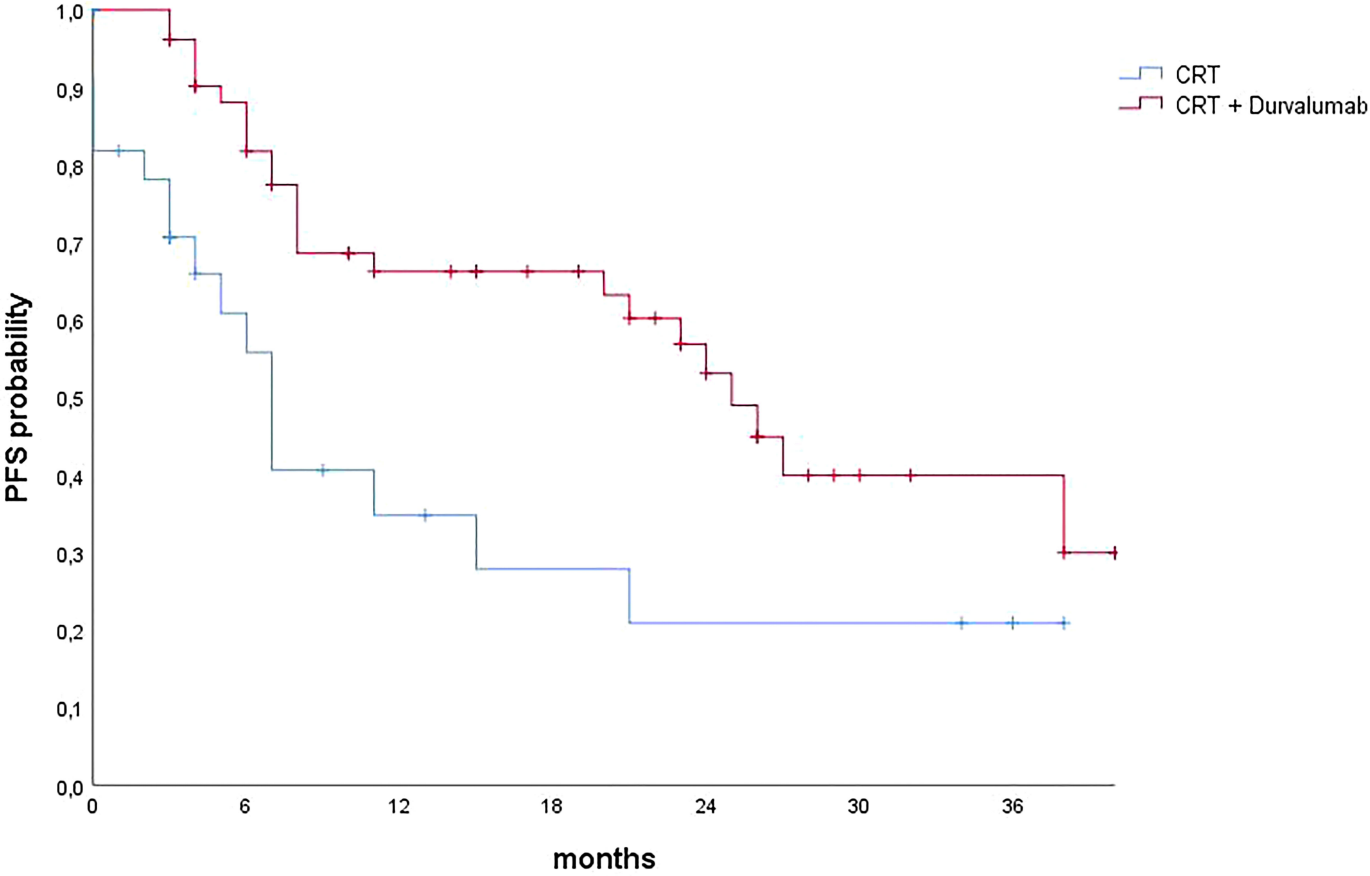
Progression-free survival curves calculated using the Kaplan–Meier method. CRT, chemoradiotherapy; PFS, progression-free survival.

The addition of immunotherapy maintenance confirmed a clinical benefit in terms of either OS or PFS. Median OS, 1-year OS, and 2-year OS in the group treated with durvalumab were 52 months, 82.5%, and 69.4%, respectively; in the group without durvalumab, they were 21 months, 56.2%, and 47.9%, respectively (p = 0.015). Median PFS, 1-year PFS, and 2-year PFS in the durvalumab group were 26 months, 66.8%, and 54.4%, respectively; in the other group, they were 7 months, 42.4%, and 24.2%, respectively (p = 0.007) (**Figures 1**, **2**).

In the group without durvalumab, excluding patients who progressed or died after CRT, the median OS and PFS were 39 and 16 months, respectively. One- and 2-year OS rates were 62.8% and 62.8%, respectively; 1- and 2-year PFS rates were 57.8% and 38.5%, respectively. These findings did not reach statistical significance when compared with the group of patients who received durvalumab.

### Adverse events

During CRT, 39 patients (45.9%) had G1-2 esophagitis. No esophagitis of G3-4 events were reported.

After CRT, 27 patients experienced lung toxicity (pneumonitis or pneumonia), and it was the most frequent AE reported. Two patients presented a G3-4 AE pneumonitis/radiation pneumonitis. The second most frequent AE reported was endocrinological alterations (five patients, 9.6%) (**Table 3**).

**Table 3:** Adverse events (AEs) reported according to CTCAE v. 5.0.

		RCT N	RCT+durvalumab N	Total N (%)
Lung toxicity				
Pneumonitis or radiation pneumonitis*		6	16	22 (25.9)
**Pneumonia**		2	1	3 (3.5)
**Other**		0	2	2 (2.4)
Lung toxicity grade				
G1		1	7	8 (9.4)
G2		3	9	12 (14.1)
G3		2	3	5 (5.9)
G4		1	0	1 (1.2)
G5		1	0	1 (1.2)
Endocrinological alterations				
G2		0	4	4 (7.7)
G3		0	1	1 (1.9)
Gastrointestinal				
G3		0	2	2 (3.8)
Hematological				
G3		0	1	1 (1.9)
Cutaneous				
G1		0	2	2 (3.8)
G2		0	2	2 (3.8)
G3		0	1	1 (1.9)
Osteoarticular				
G2		0	3	3 (5.8)

RCT, randomized clinical trial; CTCAE, Common Terminology Criteria for Adverse Events.

*Pneumonitis includes acute interstitial pneumonitis, interstitial lung disease, pneumonitis, and pulmonary fibrosis.

## Discussion

Although RCTs remain the gold standard to generate evidence to change the SoC, they often do not represent real-world clinical practice due to the highly selective inclusion criteria and the applicability after regulatory body approval.

This has led to the necessity to consider the use of real-world data (RWD) and real-world studies (RWS) to confirm the benefits or risks of a new medical product (13). After the PACIFIC trial publication, several data have confirmed that durvalumab has changed the clinical scenario of unresectable NSCLC stage III (6, 9, 14–16).

This retrospective, single-center study on 85 patients, with 52 treated with durvalumab, represents a fairly large experience compared to other single-center reports present in the literature (range 21–83 patients) (17–23).

Compared to the PACIFIC trial, this analysis showed some differences in the selected population. Patients’ median age was higher than in PACIFIC trial one (68 *vs.* 64 years), and the majority of patients were current smokers (50.6% *vs.* 16.4%). Stage IIIC was more represented (8.2% *vs.* 2.4%), and eight patients were treated for post-surgical locoregional relapse (data collected and analyzed in a multicentric series) (24). Finally, only a minority group received sCRT, which was not allowed in the PACIFIC trial, but PACIFIC-6 and GEMSTONE-301 are recently published trials that show the benefit of maintenance immunotherapy even after sCRT (25, 26). Durvalumab consolidation started, when indicated, after a longer median time (47 *vs.* <42 days). These differences could be mainly due to management issues (such as waiting lists) and clinical reasons (like slow toxicity resolution).

Despite these differences denoting a negatively selected population, similar results to the PACIFIC study were obtained for tumor response after CRT. On the contrary, PD after CRT was 9.4% in this series and 2.6% in the PACIFIC trial. Moreover, in patients treated with durvalumab, 1-year OS was 82.5% (83.1% in the PACIFIC trial), and 2-year OS was 69.4% (63.3% in the PACIFIC trial). One-year OS for patients who did not receive durvalumab was lower than in the placebo arm in the PACIFIC trial (56.2% *vs.* 74.6%). In the same group, the 1-year PFS was 42.4% *vs.* 35.3% of the PACIFIC (6).

These results could be partly explained by the fact that in the PACIFIC trial, patients were randomized to durvalumab or placebo exclusively after demonstration of not progressed disease after CRT. Therefore, patients with PD after CRT were excluded from the trial. In the present analysis, patients who progressed after CRT have been also included in the survival analysis. This aspect could be considered a sort of methodological deviation within the study. However, this work did not expect to faithfully replicate the PACIFIC trial but wanted to carry out a global evaluation of patients treated with radical intent for unresectable stage III NSCLC. Nevertheless, after excluding from the analysis patients who died or progressed after CRT, PFS and OS still improved in the durvalumab group despite no statistical significance. This result could be explained by the limited number of censored events and the surprising performance of patients treated without durvalumab.

In this series, 33 patients (38.8%) did not start durvalumab. Among these, 13 patients had negative levels of PD-L1 expression. In the PACIFIC trial, the benefit in terms of OS and PFS was detected in all the subgroups of PD-L1 expression in the durvalumab arm, except for OS in patients with PD-L1 expression less than 1%. These specific data, extracted from a *post hoc* analysis, led the European Medicines Agency (EMA) to approve the maintenance with durvalumab only for cases with PD-L1 expression higher than 1%. Furthermore, in clinical practice for patients with basal PD-L1 expression of less than 1%, a re-biopsy after CRT in order to re-test PD-L1 expression could be considered as an option. In fact, it is assumed that CRT can induce changes in the tumor microenvironment and, consequently, in the expression of PD-L1 (27). In this regard, two patients presented a PD-L1 expression higher than 1% after re-biopsy following CRT, so they were started on durvalumab.

In this study, patients presented good compliance to immunotherapy and developed toxicities in line with the results of the RCT and RWD. Pulmonary toxicity (all grades) was observed in 31.8% of patients, and grade 3 was minimal (3.8%), just like in PACIFIC (33.9%—G3 3.4%) and other RWDs (35%—G3 6%) (4, 9). This good compliance allowed patients to continue immunotherapy; in fact, in our study, only 11.5% of patients discontinued the maintenance program due to toxicity. In the PACIFIC trial, these data were reported in 15.4% of patients.

Though 87% of patients underwent a concurrent regimen of CRT, grade 2 acute esophageal toxicity occurred in 25.9% of the population and none of grade 3 or higher. Furthermore, patients included in this analysis had worse clinical features (such as age, COPD, and smoke status) and higher stages of disease than patients included in RCTs.

These data could probably suggest that, with accurate clinical support (prevention and management of toxicities or pulmonary rehabilitation) and the use of modern radiotherapy techniques, even fragile patients could aspire to treatment with curative intent (28–31).

The largest real-world study is surely PACIFIC-R, which enrolled 1,399 patients in 11 countries. This is an international, retrospective study of patients who started durvalumab within an early access program between September 2017 and December 2018 (16).

Notably, the OS and PFS reported in PACIFIC-R are similar to those in the current series. Instead, the all-grade pneumonitis rate is lower.

A comparison of clinical and toxicities outcomes among the PACIFIC trial, PACIFIC-R study, and the current series is summarized in **Table 4**. It should be noted that these three studies have some inherent differences, such as overall maintenance immunotherapy time (PACIFIC-R allowed durvalumab even beyond 1 year) and start date for calculating survival and FU (randomization date for PACIFIC, initiation of durvalumab for PACIFIC-R, and end of radiotherapy for ongoing series).

**Table 4:** Comparison among current series (excluding patients who progressed or died after CRT) and PACIFIC trial and PACIFIC-R.

		Current series		PACIFIC trial		PACIFIC-R
		CRT	CRT+durvalumab	Placebo	Durvalumab	Durvalumab
**Time between end of RCT and start of durvalumab (days)**		–	47	–	–	56.0
**Median FU (months)**		20.0		34.2		23.5
**OS**	1 year (%)	62.8	82.5	74.6	83.1	–
	2 years (%)	62.8	69.4	55.3	66.3	71.2
	Median (months)	39	52	29.1	47.5	NR
**PFS**	1 year (%)	57.8	66.8	34.5	55.7	62.2
	2 years (%)	38.5	54.4	25.1	45	48.2
	Median (months)	16	26	5.6	16.9	21.7
**Pneumonitis any grade**		18.8	30.7	24.8	33.9	17.9

CRT, chemoradiotherapy; RCT, randomized clinical trial; FU, follow-up; OS, overall survival; PFS, progression-free survival.

This work describes a monocentric, large, and homogeneous experience of patients treated with radical treatment for unresectable stage III NSCLC. As foreseeable, the selection of patients and the treatment conditions were slightly less favorable than the registration study. However, globally, patients were properly identified, and the clinical results were in line with the reference study and other similar experiences.

Unfortunately, due to the shorter follow-up, this experience is unable to evaluate the 5-year OS, which represents one of the major strengths of the PACIFIC trial. This RWS, like others, is useful to consolidate the data obtained from the PACIFIC trial and can be used to investigate still open issues, as the role of durvalumab in patients with oncogene-addicted NSCLC and in patients with controlled autoimmune diseases and the choice of treatment after progression to durvalumab, including local ablative therapies if oligometastases are evident.

Currently, real-world data on the use of durvalumab for unresectable NSCLC III stage confirm the safety and efficacy of this treatment in an evolving scenario. Indeed, recent new drugs, such as monalizumab, oleclumab, and sugemalimab are appearing as a potential alternative for maintenance after CRT (26, 32).

The introduction of durvalumab after CRT in stage III NSCLC has changed the standard of care. The data reported in this clinical scenario show that durvalumab as maintenance has an acceptable toxicity and a favorable efficacy, supporting the use of this therapeutic strategy with curative intent by recommending an accurate selection of the patient and his/her management within a multidisciplinary team.

## Data availability statement

The raw data supporting the conclusions of this article will be made available by the authors, without undue reservation.

## Ethics statement

The studies involving human participants were reviewed and approved by Ethics committee of Brescia. Written informed consent for participation was not required for this study in accordance with the national legislation and the institutional requirements.

## Author contributions

All the authors have equally contributed in conceptualization, analysis, evaluation, investigation, data curation and in writing this research paper. All authors contributed to the article and approved the submitted version.
