# Supplementary material for: Unresectable stage III non-small cell lung cancer: could durvalumab be safe and effective in real-life clinical scenarios? Results of a single-center experience
Source: Front Oncol. 2023 Jul 4;13:1208204. doi: 10.3389/fonc.2023.1208204 (PMC10352832; doi:10.3389/fonc.2023.1208204)
Supplement: Supplementary file 1 [file Table_1.docx]

**Table S1.** Tumor response after CRT according to Response Evaluation Criteria in Solid Tumors [RECIST], version 1.1.

|  |  |  |
| --- | --- | --- |
| **Tumor response** | **N** | **(%)** |
| **Complete response** | 2 | 1.2 |
| **Partial Response** | 41 | 48.2 |
| **Stable Disease** | 31 | 36.5 |
| **Progression Disease** | 8 | 9.4 |
| **Not evaluated** | 3 | 3.5 |
